# Rapid Risk Assessment Framework to Estimate Potential for Spillback at Human–Wildlife Interfaces

**DOI:** 10.1155/tbed/4334954

**Published:** 2025-06-13

**Authors:** Travis McDevitt-Galles, Tricia L. Fry, Katherine L. D. Richgels, Daniel A. Grear

**Affiliations:** National Wildlife Health Center, U.S. Geological Survey, Madison, Wisconsin, USA

## Abstract

More than 60% of emerging infectious diseases of humans have a wildlife origin, and when these diseases spread through human populations to new geographical areas, there is a considerable risk of spillback from humans to wildlife species. Spillback events can have severe consequences for wildlife populations, where the disease may cause morbidity and mortality, and human populations, where the establishment in wildlife may lead to prolonged transmission or new exposures in humans. Mitigating these consequences requires identifying the key risk factors that lead to human–wildlife transmission events and implementing risk-reducing actions, a challenge given that cross-species transmission events are rare and often data deficient. To identify potential species and locations that are most likely to lead to these rare events, we developed a spatially explicit, rapid risk assessment framework that incorporates three components of the spillback process: wildlife susceptibility, wildlife exposure, and pathogen introduction pressure. To demonstrate the broad applicability of our framework, we conducted a rapid risk assessment on two recent emerging zoonotic pathogens in humans, severe acute respiratory syndrome coronavirus 2 (SARS-CoV-2) and mpox, to determine the relative spillback risk to wild mammalian species in the continental United States. The rapid risk assessment identified both species and locations with higher than expected spillback risk, providing managers and researchers with valuable information to prioritize surveillance and risk-mitigation actions. Our framework represents a rapid and flexible approach to assess the risks of spillback to wildlife populations during rapidly evolving zoonotic disease outbreaks.

## 1. Introduction

The global transmission of emerging zoonotic diseases in humans has led to concerns regarding the spread of these diseases from humans into novel wildlife populations, a disease transmission process known as spillback [1–3]. Spillback events that lead to disease establishment in novel reservoir hosts add complexity for control efforts and increases the likelihood of pathogens infecting susceptible human populations (a process called spillover; [4, 5]). Furthermore, infections in wildlife can lead to possible viral recombination events [6, 7] and pathological outcomes for wildlife, resulting in conservation concerns. For example, spillback dynamics have been identified as playing a major epidemiological role in the spread and maintenance of bovine tuberculosis around the world [8]. With spillback events from the native cervid population back to cattle being the main pathway of bovine tuberculosis infection in North America [9, 10]. A major barrier to our understanding of these processes is capturing data on spillback transmission events, which may be rare in nature and occur in wildlife populations with little or no systematic monitoring [11]. This paucity of information restricts our ability to make empirically based inferences regarding the drivers of human–wildlife disease transmission and implement mitigations to reduce the spillback risk [12].

A major obstacle in obtaining the necessary data to improve our understanding of the dynamics of spillback events is that these events are rare and short-lived [13–15]. Resulting in a limited window to collect and analyze data and respond at the wildlife–human interface. In addition, the timing of these transmission windows often overlaps with major public health emergencies that demand substantial economic and public-health resources [16, 17]. During these windows, developing and implementing targeted wildlife sampling efforts to capture potential spillback events are often deprioritized [18], thereby missing the opportunity to acquire data needed to gain insight into the spillback process. As such, prioritizing sampling efforts by restricting either the targeted species or geographic range based on knowledge of one or more components of the spillback process could greatly improve the chances of detecting these adverse events [19, 20]. To effectively sample wildlife populations and improve our understanding of the spillback process during these brief transmission windows, it is critical to have a flexible and simple framework that can rapidly produce guidelines to help focus sampling efforts on species and geographic locations that have a relatively higher risk of spillback events occurring.

**Figure 1:**
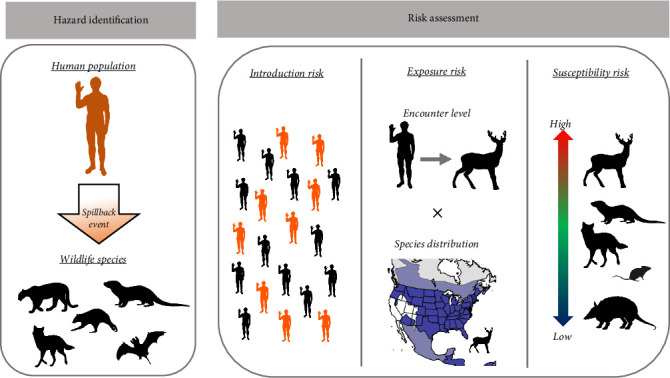
Our primary identified hazard is a risk of spillback events, the spread of infectious diseases from humans into wildlife populations. We have identified three key risk factors that will influence our model. (1) Introduction risk or an estimate of infectious agents at a given location. (2) Exposure risk that estimates contact levels between wildlife and humans, as well as the spatial distribution of wildlife species. (3) The susceptibility of different wildlife species to pathogens.

To rapidly assess the risks of spillback, we used a simplified conceptual risk model that can be applied broadly to outbreaks that are data sparse and/or have high uncertainty. While our understanding of the specific mechanistic processes and core variables driving these dynamics is limited, previous research on spillover events has identified three core phases that determine the likelihood of cross-species transmission [14]. The first phase is the *introduction risk*, the amount of a pathogen in the environment [21]. The second phase, *exposure risk*, is driven by wildlife distributions and behavior traits shaping wildlife exposure to the pathogen [22, 23]. The final phase, *host susceptibility*, is the likelihood that a given wildlife species becomes infected given exposure, which is primarily driven by genetic and immunological factors [24]. By breaking the spillback processes into three discrete phases, we can begin to identify and group established risk factors to develop a broad and informative framework for evaluating initial spillback risk (Figure 1). By combining these three components we developed a rapid risk assessment framework that can identify species and locations with an elevated relative risk of the occurrence of spillback events. We performed a rapid risk assessment of spillback risk across wildlife species and geographic locations for two global pandemic-causing viruses, the severe acute respiratory syndrome coronavirus 2 (SARS-CoV-2) and mpox (Orothopox genus), to demonstrate the usefulness and flexibility of this framework.

SARS-CoV-2 is a zoonotic pathogen that causes COVID-19 in people. Since late 2019, this virus has infected more than 660 million people and caused more than 6.5 million deaths worldwide [25, 26]. SARS-CoV-2 is primarily transmitted from human to human through contact and inhalation of infectious aerosolized particles emitted from infected individuals or through contact with infected environmental particles [27]. While specific transmission routes associated with human-to-wildlife transmission have not been fully elucidated, there have been several confirmed spillback events associated with animal species with close contact with humans. This includes wild cervid populations [23], big cat species in zoos [28], domestic and free-ranging mink [29, 30], and companion animals [31].

The Mpox virus is considered the most concerning orthopoxvirus for humans since the eradication of smallpox [32]. Mpox was initially described in 1958, and since then, there have been numerous pandemics of various magnitudes [33]. The most recent global pandemic of mpox started in early 2022, with the first confirmed case in the United States in May of that year [34]. Between May and October 2022, all states within the United States reported mpox infections, with peak infections in the United States occurring in early August. During this period there were over 30,000 confirmed cases in the United States [35]. While the location and source of initial spillover events to humans have not been identified, there is a large diversity of rodent species that serve as potential reservoir hosts for mpox [36, 37].

Both of these viruses are zoonotic, with a broad pool of suspected and potential wildlife reservoirs [38–40], increasing the potential for spillback events. Our rapid risk assessment, which incorporates currently available knowledge about introduction risk, exposure risk, and host susceptibility, provides an initial assessment of high-risk species and locations for these two pathogens to help develop targeted sampling efforts. Our model framework results in actionable information for designing surveillance systems and targeted wildlife sampling; furthermore, it is highly flexible, allowing for the inclusion of updated information to improve future risk assessment results.

## 2. Methods

### 2.1. Risk Assessment Framework

We used a risk model framework following the risk identification and assessment process adopted by the World Organization of Animal Health [41], which produces *relative* risk scores with higher values indicating a greater relative likelihood of a hazard occurring. We defined the hazard, the harmful outcome of the event, as the spillback of a pathogen from human to wildlife. The estimated risk of this hazard is the combination of three components*: introduction risk*, *host exposure*, and *host susceptibility* (Equation (1); [11, 14]). We defined *introduction risk* as a location-level trait based on a function incorporating human population size and infection rate. We defined *host exposure* as the product of the amount of wildland–urban interface (WUI) and the occurrence of a given wildlife species in the specific area, with the assumption that species with higher wildlife–urban interfaces would lead to increased risk of spillback. *Host susceptibility* is a species-level trait and represents the likelihood of a wildlife species being infected by the pathogen given exposure. We estimated host susceptibility using a rank variable based on a literature review of studies quantifying host susceptibility to the relevant pathogen. We developed a simple risk assessment model that allows us to combine the components driving spillback events while making minimal assumptions about the underlying biological processes. We estimated spillback risk at the species by county level by using an additive approach for our spatial components (introduction pressure and exposure risk) and multiplied this value by the species susceptibility score. While the specific risk score is unitless, we can compare relative risk across species and space by summing risk across the desired units (species or spatial units, i.e., counties or states). For both SARS-CoV-2 and mpox viruses, we restricted our spillback risk assessment to mammalian species since neither virus has been detected outside the mammalian order, occurring in the continental United States with the finest spatial unit being the county or county equivalent level to inform introduction and exposure risk since these data are readily available, that is, human population levels and infection data.

(1) $$\begin{array}{c} {\text{Spillback risk score}}_{j,i}=\left({\text{introduction}}_{j}+\;{\text{exposure}}_{i,j}\right)\times \;{\text{susceptibility}}_{i}j=\text{location identiy},i=\text{species identiy}. \end{array}$$

### 2.2. Introduction Risk

Introduction risk is a proxy of infectious agent pressure at a geographic location. How introduction risk is defined should be pathogen-specific and may be limited based on data availability or quality but should represent the infection agent pressure originating from the source species (humans for a spillback hazard or wildlife for a spillover hazard). In our SARS-CoV-2 model, this is the product of the county's human population and SARS-CoV-2 infection rate. SARS-CoV-2 infection rates were monitored at many levels, but we used county-level estimates as they were the smallest spatial scale that was widespread and readily accessible. County-level populations were obtained using the 2020 census data [42]. For SARS-CoV-2 infection levels, we fit a Bayesian generalized additive model (GAM) using the Rstanarm package [43] to weekly infection rate data (per 100 k) spanning from March 3, 2022 to August 4, 2022 [26]. We then quantified the area under the curve of the best-fitted trend line to represent the cumulative infection levels across time for a given county; we then multiplied this value by the county's population size to get the spatially explicit risk of introduction over this same period.

For mpox, we estimated introduction risk as the product of the per-person infection level at the state level and the county's total population. Currently, we only have access to cumulative state case counts of the mpox clade II subtype up until January 10, 2024, which prevents us from using a similar approach used to quantify introduction risk for SARS-CoV-2.

### 2.3. Exposure Risk

Exposure risk captures the potential for a wildlife species to interact with an infectious agent. How we define exposure risk in our risk assessment will be dependent on our definition of introduction risk, as the type of introduction pressure will determine what the exposure landscape looks like. For our risk assessment, we defined exposure risk based on the total amount of WUI in a given county, *j*, conditional on the occurrence of species *i*, in county *j* (Equation (2)). WUI is defined as the area where human development meets or is entangled with undeveloped wildlands and captures the relative amount of human–environment conflict [44]. As spillback transmission will likely require close wildlife–human contact and the mechanisms that drive such contact for SARS-CoV-2 and mpox are unknown, WUI represents a simple and readily available metric for our rapid risk assessment. Our county-level metric of human development and wildland interface was based on updated estimated WUI values obtained from the SILVIS lab web portal [44].

(2) $$\begin{array}{c} {\text{Exposure}}_{i,j}={\text{WUI}}_{j}\times {\text{species occurrence}}_{i,j}. \end{array}$$

Species occurrence was defined at the county level and was treated as a binary variable with a value of 1 indicating species presence in a county and 0 if absent. We obtained county-level mammal species occurrence data using published range maps obtained from the International Union for Conservation of Nature (IUCN) database [45]. We overlayed the species range maps with county-delimited maps and determined occurrence based on if the county area intersected with the species range using the SF package [46]. As we currently lack knowledge of the infection pathways for SARS-CoV-2 and mpox, we treated exposure to risk as the same for both pathogens.

### 2.4. Host Susceptibility

Host susceptibility captures the likelihood of a wildlife species becoming infected given an exposure to the pathogen. To quantify species-specific host susceptibility to SARS-CoV-2, we compiled 11 studies that either estimated theoretical susceptibility based on species variation across the structure of their angiotensin-converting enzyme 2 (ACE2) or used machine learning approaches to estimate species level susceptibility. By including papers that use machine learning techniques, we are able to expand the coverage of estimated mammal susceptibility to over 5000 species [24]. The ACE2 enzyme is the primary binding agent for the SARS-CoV-2 spike protein [47, 48], and variation in the structure is thought to convey variation in susceptibility to infection [3]. We compiled the studies by searching Google Scholar and Web of Science using the following keywords: “SARS-CoV-2,” “wildlife,” “susceptibility,” and “ACE2.” We restricted our search to papers that were made available (either published or as a preprint) by December 2021. For each of the 11 studies, we grouped each species into one of three susceptibility groups (high, medium, or low susceptibility) based on the authors' interpretation and classification of susceptibility. We then aggregated across all studies to estimate a species-specific susceptibility grouping (high, medium, or low) based on how the majority of studies identified susceptibility risk. If, for a given species, there was a lack of a majority agreement across studies, we assigned it to the “medium” group. Any wildlife taxa that did not have an associated susceptibility score were removed from the SARS-CoV-2 risk assessment model.

The current knowledge of wildlife susceptibility to mpox infection is largely unknown, especially for native species in the United States [49–51]. Furthermore, there exist very few experimental infection studies, and no study that we are aware of that uses species variation in cellular structure (e.g., ACE2 studies for SARS-CoV-2) to estimate species susceptibility. To work with this large uncertainty, we assigned only two values for our species' susceptibility to mpox ranking, high susceptibility, which was assigned to any taxa that are considered to be susceptible to mpox based on published work, and low susceptibility was assigned to all other taxa. Using only two rankings allowed us to elevate taxa with known susceptibility while not having to distinguish taxa with little to no data between medium and low susceptibility levels.

To quantify the categorization of host susceptibility in our risk assessment, we assigned a value of 1.00 to species in the “high” category, 0.50 for the “medium” and 0.25 for species in the “low” category. We used this range value to conservatively capture the differences in theoretical susceptibility while not treating the categorization as established knowledge (i.e., assuming that low susceptible species are incapable of becoming infected).

### 2.5. Sensitivity Analysis

To determine how each of the core variables (species susceptibility, exposure, and introduction) impacted the species and county-level spillback risk rank, we re-calculated the risk assessment scores for both SARS-CoV-2 and mpox while varying the weight of these distinct variables and ranked species and counties. We used a Latin hypercube sampling (LHS) approach to explore how varying the weight of each of the different components over a uniform distribution between 0 and 2 shifted the relative species and county ranks. We used the LHS package [52] in the R statistical language [53] to divide the parameter spaces into 1000 equal-probability intervals, randomly select a weight value in each interval, and created 1000 parameter sets by combining the weight values of each parameter. We assessed sensitivity by comparing the rank-order of risk scores between counties and species to the rank-order with all weights equal to 1 by calculating a Pearson's correlation coefficient. We analyzed the relative contribution of the different spillback components to variance in species and county rankings by estimating the partial rank correlation coefficients for each spillback component.

To assess how sensitive our results are to *how* we define our variables for the SARS-CoV-2 and mpox risk assessment, we reran our assessments using variations in how we define the metrics for each core component. For each alternative metric, we kept the other two components consistent with how we initially defined them (Table 1). After running the risk assessment with the alternative metric, we compared the new ordinal rankings of species and county spillback risk with the newly estimated rankings. By focusing on rank instead of risk value, we compared output across the different risk assessments. We assessed the sensitivity of our parameter definitions by visually comparing the deviations in rank from our baseline component definitions. In our qualitative assessment, large deviations in rank from the alternative models compared to the baseline would be indicative that the component is highly sensitive to how we defined it; small deviations in rank from the alternative models compared to the baseline suggest that the component is less sensitive to our definition.

## 3. Results

**Figure 2:**
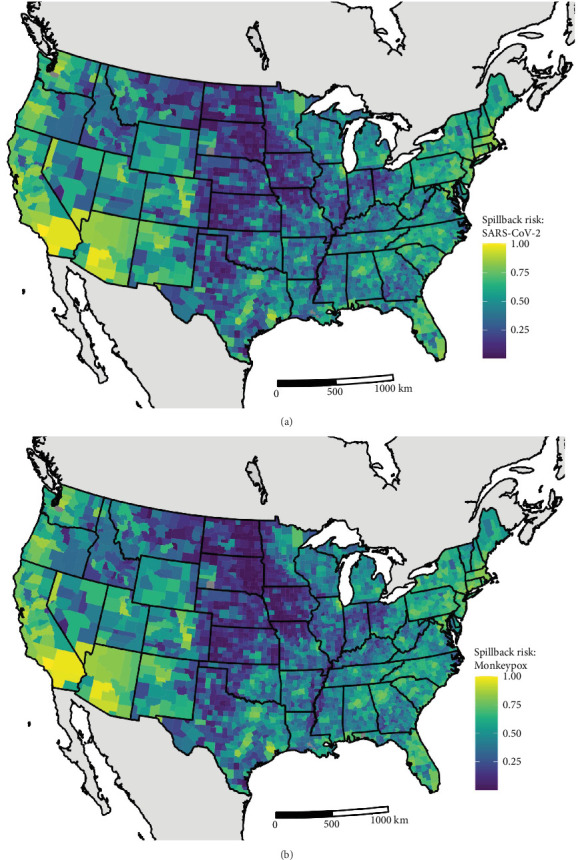
(A) Spatial variation in county level SARS-CoV-2 wildlife spillback risk. (B) Spatial variation in spillback risk for monkeypox. Warmer colors indicate higher relative spillback risk ($\text{County}\;\text{spillback}\;{\text{risk}\sim \;\text{score}}_{j}=\sum_{n=1}^{i}\left({\text{introduction}}_{j}+{\text{exposure}}_{j}\right)\times {\text{suscept}}_{i}$). Spillback is the spread of infectious diseases from humans into wildlife populations. Basemaps are from the US Department of the Census Date county boundary files and the Natural Earth Dataset.

**Figure 3:**
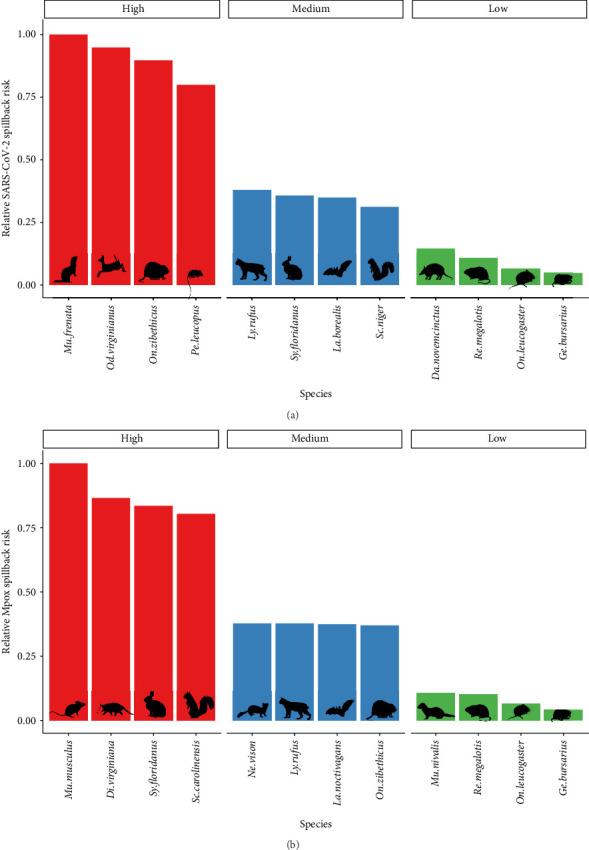
Relative spillback risk ranked by species for (A) SARS-CoV-2 and (B) mpox. Species-level spillback risk was quantified by summing the total risk across the species range. Spillback risk was scaled relative to the highest total risk score. Bars are colored based on relative risk grouping. High-risk taxa are in the 75th percentile of species, medium taxa are between 26th and 74th, low-risk taxa are in the 25th percentile of species. The taxa displayed are a subset of the total taxa quantified. Spillback is the spread of infectious diseases from humans into wildlife populations. *M. frenata*, *O. virginianus*, *O. zibethicus*, *P. leucopus*, *M. pinetorum*, *M. musculus*, *D. virginiana*, *S. floridanus*, *S. carolinensis*.

We quantified the relative spillback risk of 291 mammal species across the contiguous 48 states for two pandemic-causing viruses, SARS-CoV-2 and mpox. By aggregating risk to the species and county level we can identify both locations (Figure 2) and species (Figure 3) of high risk of spillback events. For spatial variation in SARS-CoV-2 spillback risk, we observed that counties located in the southwestern United States had the highest total risk score, with nine out of the top 10 counties being in either California (6/10), Arizona (2/10), or Nevada (1/10) (Figure 2A). Across the Continental United States, we observed several highly ranked relative risk areas situated in areas surrounding high-population urban cities, such as Miami-Dade, FL; Houston, TX; Boston, MA; and Chicago, IL. Across species, we identified the long-tailed weasels (*Mustela frenata*) as having the highest aggregated risk, followed by white-tailed deer (*Odocoileus virginianus*), muskrats (*Ondatra zibethicus*), and white-footed mice (*Peromyscus leucopus*) and woodland vole (*Microtus pinetorum*). Species within the 75th SARS-CoV-2 spillback risk quantile are largely dominated by the orders Carnivora and Rodentia, which make up 50% (8/16) and 44% (7/16) of the species, respectively.

For mpox, we estimated similar patterns to SARS-CoV-2 in the spatial variation of spillback risk, with the highest total risk scores occurring in the southwestern United States and areas with high human density in the northeast and south Florida (Figure 2B). For species, we identified the common house mouse (*Mus musculus*), the Virginia opossum (*Didelphis virginiana*), the eastern cottontail (*Sylvilagus floridanus*), and the eastern gray squirrel (*Sciurus carolinensis*) as species most likely to facilitates spillback events. Species within the 75th mpox spillback risk are largely dominated by the orders Rodentia and Carnivora, making up 66% and 17% of the species, respectively.

### 3.1. Sensitivity Analysis

**Figure 4:**
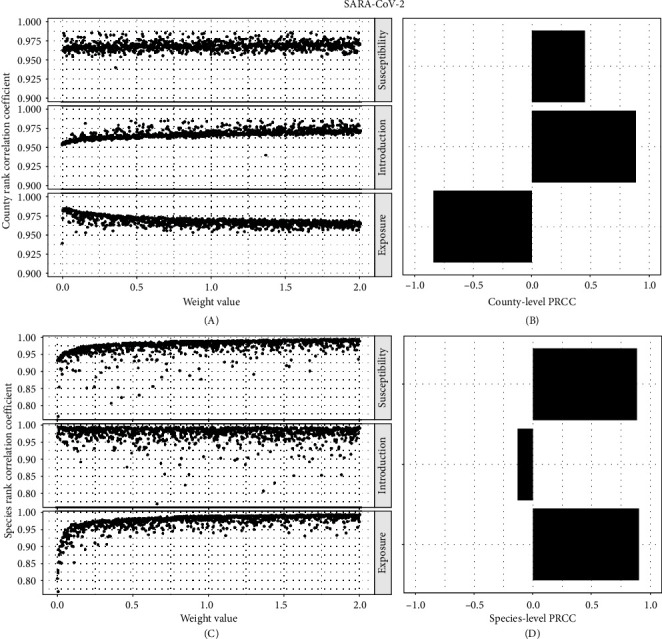
Risk component sensitivity analysis for SARS-CoV-2. Marginal Pearson's correlation coefficient's of (A) county risk rank and (C) species risk rank using a Latin hypercube sampling design of varying susceptibility, exposure, and introduction risk components. Along with corresponding partial rank correlation coefficients (PRCC) of simultaneously varied risk component weights for (B) county and (D) species levels.

We explored two distinct aspects of sensitivity in our risk assessment, across and within the component. For the across-component analysis of SARS-CoV-2, the ranking of county risk scores was overall insensitive to weight parameters with mean Pearson's rank correlation coefficient 0.968 (range: 0.954–0.985) compared to all parameter weights equal to 1 (Figure 4A). The weight on susceptibility and introduction resulted in positive sensitivity to overall risk; that is, lower weights resulted in more variation in the rank order of county risk, and higher weights resulted in a very similar rank order compared to assigning weights of 1 to all risk components (Figure 4B). Varying weights on exposure risk resulted in negative sensitivity, indicating that lower weights on exposure risk generated more similar county rank-order. There was also a high mean of Pearson's rank correlation coefficient for species SARS-CoV-2 risk (mean = 0.97; range: 0.81–0.99) (Figure 4C). The weight assigned to susceptibility and exposure risk generated positive sensitivity to overall risk, and the introduction risk weight generated a small negative sensitivity for the overall species risk rank-order (Figure 4 D).

**Figure 5:**
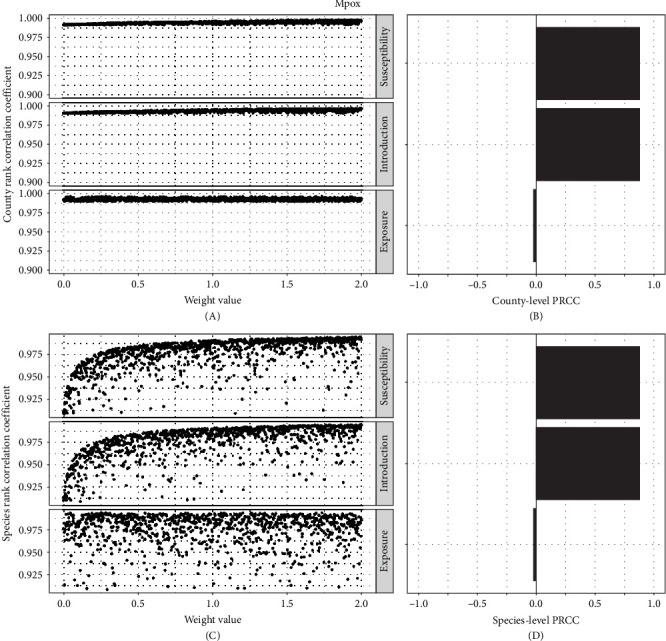
Risk component sensitivity analysis for mpox. Marginal Pearson's correlation coefficient's of (A) county risk rank and (C) species risk rank using a Latin hypercube sampling design of varying susceptibility, exposure, and introduction risk components. Along with corresponding partial rank correlation coefficients (PRCC) of simultaneously varied risk component weights for (B) county and (D) species levels.

The ranking of county risk scores for mpox was relatively insensitive to weight parameters with mean Pearson's rank correlation coefficient 0.99 (range: 0.991–0.997) compared to all parameter weights equal to 1 (Figure 5A,B). There was also a high mean of Pearson's rank correlation coefficient for species (mean = 0.97; range: 0.90–0.99) (Figure 5C). The weight on susceptibility and introduction risk generated positive sensitivity to the rank-order of county and species overall risk, while the exposure weight had no impact (Figure 5D).

**Figure 6:**
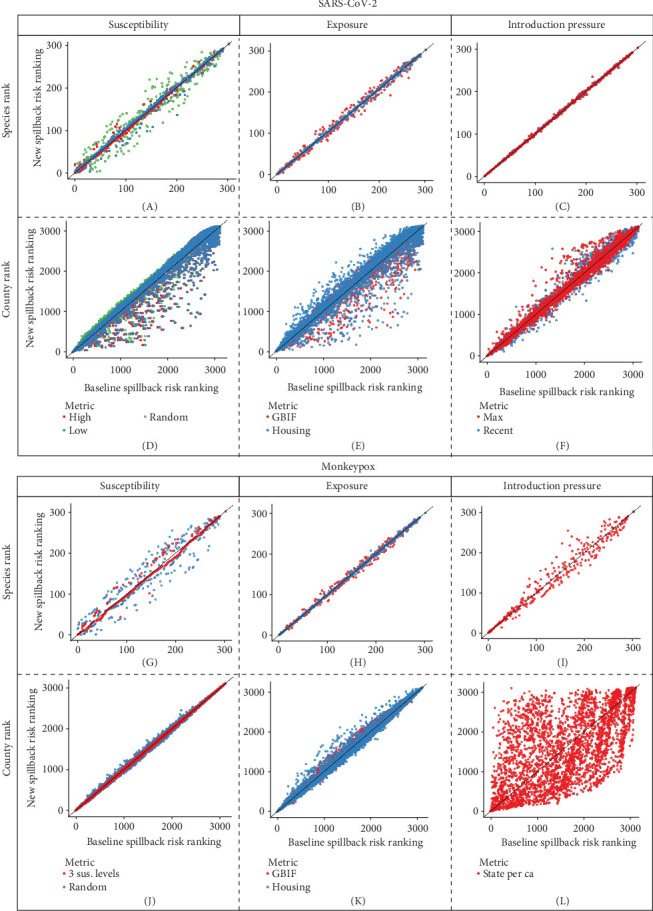
Within component sensitivity analysis for (A–F) SARS-CoV-2 and (G–L) mpox species and county-level spillback risk. Spillback is the spread of infectious diseases from humans into wildlife populations.

For SARS-CoV-2 at the species rank level, we saw the most variation in rank and, thus, sensitivity in the species susceptibility component relative to the other two components (Figure 6A–C). At the county level, we observed large sensitivity across all three components, with the highest level of sensitivity occurring in the exposure and susceptibility components (Figure 6D–F). For mpox at the species rank level, we observed a large variation in relative rank at both the species susceptibility and introduction risk components with minimal variation at the exposure component (Figure 6G–I). At the county level, we observed a large variation at the introduction component level with a moderate level of variation at the exposure level (Figure 6J–L).

## 4. Discussion

By developing a simple, informative risk assessment framework for estimating spillback potential, we provide a valuable tool for improving our ability to detect, understand, and respond to pathogen spillback. The current framework of our risk assessment uses readily available information related to introduction risk, exposure risk, and host susceptibility to derive estimates of relative spillback risk. This results in a rapid and highly flexible risk assessment model. As demonstrated, this model can be used to develop prioritization-based surveillance strategies and reduce the current data limitation gaps, both of which improve our understanding into the processes shaping spillback events.

In our SARS-CoV-2 risk assessment, we identified relative spillback risks across various species and spatial locations, which land use managers can use to inform actions aimed at mitigating these risks. The wildlife species with the highest risk scores exhibited a combination of medium-to-high susceptibility and extensive geographical ranges overlapping with human-populated areas. Notably, some of our top species, such as white-tailed deer, raccoons, American opossums, and white-footed mice, have strong associations with human-modified landscapes [54]. However, the current risk assessment framework does not incorporate species-level data on potential human interactions. Future iterations of this framework could benefit from including this data, which would likely elevate the risk scores for these taxa and others closely associated with humans. Additionally, several high-risk taxa, including white-tailed deer and white-footed mice [55], have already been suspected or confirmed to be infected with SARS-CoV-2 due to spillback events. While the specific exposure pathway for these specific spillback events remains unknown, the most likely pathway is thought to be through direct contact with infected humans from cohousing (white-footed mice) or from farming/hunting (white-tailed deer). Spatially, we observed higher spillback risks in areas characterized by larger population sizes—often correlated with elevated infection levels—and significant wildlife–urban interfaces, particularly in the Southwestern and Northeastern regions of the United States. These findings align with our conservative approach to estimating spillover pathways, as we would expect heightened spillback risks in areas with large populations and moderate-to-high interaction potential. A better understanding of the likely spillback transmission pathways for SARS-CoV-2 (e.g., rehabilitation centers, feeders, outdoor recreation) could yield a more nuanced spatial distribution of spillback risk. Achieving this would require increasing complexity in both the introduction pressure and encounter risk components of our risk assessment model.

Our mpox spillback risk assessment has produced an initial quantification of species-specific and location-specific risks that can help guide surveillance efforts to improve our understanding of potential mpox reservoir hosts within the United States. Currently, our knowledge of likely spillback hosts for mpox in the United States is limited, representing a significant gap in our ability to monitor and predict potential outbreaks [56]. The species identified by our model as having the highest spillback risk include taxa previously recognized as susceptible to mpox and strongly associated with urban environments, such as house mice, gray squirrels, and American opossums. Although there have been no confirmed detections of mpox infecting these taxa, their combination of susceptibility and potential exposure through interactions with infected humans in urban areas makes them high priorities for mpox monitoring in wildlife populations. Similar to our SARS-CoV-2 risk assessment, we observe the highest spillback risks in areas with large populations and elevated infection levels, particularly in the Southwestern and Northeastern regions. The consistent spatial patterns observed between the two pathogens are likely driven by our conservative approach in defining introduction pressure and exposure risk in our model. Incorporating additional pathogen-specific exposure routes could lead to more varied spatial patterns of spillback risk between the two pathogens.

A core strength in our risk assessment framework is its reliance on clear and simple components, efficiently obtained from open-source public health and land use databases. Defining model components is integral to the usefulness of the model; thus, it is critical to assess how we defined the different components that contribute to the overall base risk. For both SARS-CoV-2 and mpox, species-level spillback risk scores are highly sensitive to our definitions of the susceptibility component. Currently, our susceptibility estimates are defined using either theoretical susceptibility based on the ACE2 enzyme [24] or a combination of limited experimental and field-based work [50, 57], resulting in large uncertainty in these susceptibility scores [50, 58]. This, coupled with the large influence susceptibility has in determining species risk levels, highlights the need to develop broader experimental and validated metrics for species susceptibility. These “real time,” data driven, descriptions of species susceptibility, especially in the case of SARS-CoV-2, provide a foundation for the model and can be updated as additional information is obtained. Improved susceptibility data, as available, would allow us to estimate “actualized” susceptibility in our models, improving our estimates of wildlife susceptibility and reducing uncertainty.

Similarly, there are limitations in the data related to obtaining and extrapolating the spatial extent of the pathogen of interest. For SARS-CoV-2, we selected county-level risk and saw high sensitivity based on how we defined the components of exposure and introduction pressure. Developing more process-based metrics for these two components will enable researchers and managers to test the drivers of spatial variation in spillback risk. The exposure component of the model can also be expanded to include details about specific human–wildlife interfaces with high transmission potential. Such a model could include parameters such as species and county-level hunting pressure [59], location and number of wildlife species in wildlife rehabilitation centers [60], or current monitoring efforts (i.e., for conservation or management goals; [61]). Integrating user-based reporting of animal encounters or sightings, such as iNaturalist, may improve estimates of human–wildlife encounter intensity [62, 63]. Including additional information on the pathogen, such as strain type or alternative transmission pathways, and incorporating that into the introduction component (i.e., transmission through wastewater [64]) could improve estimates of introduction risk. A core challenge in implementing these additional parameters is identifying and integrating data into a national data model while maintaining appropriate spatial resolution [65, 66]. Understanding how to best integrate the risk assessment framework with new data types will be crucial as we build in complexity during future iterations.

Currently, our framework estimates the relative risk of a spillback event occurring and does not account for the likelihood that the spillback event will lead to an established infection in the population; however, our framework allows for the modification and or inclusion of other hazards or processes that might be of interest or concern to researchers and managers. One of the core concerns in the spillback process is the potential development of a novel reservoir host [67, 11]. For a spillback event to result in the establishment of a reservoir host, there needs to be persistent transmission and infection within the wild species population, as recently observed with SARS-CoV-2 and white-tailed deer [68, 69]. To better evaluate reservoir host risk in our framework, we can include additional components that capture reservoir host establishment risk, such as relevant species-level traits, including the number of virus species known to infect a species [21] and the phylogenetic distance from an established reservoir taxon of the given pathogen [58]. Including this information into the existing framework may produce risk scores that can identify reservoir host establishment risk and enable managers to take additional precautionary measures when handling or interacting with these sensitive taxa. However, integrating these additional data streams carries its own obstacles, such as variation in data coverage and uncertainty in how to best define reservoir host risk [58]. Further hazards that could be incorporated in different risk assessment iterations include but are not limited to, virus recombination risk [62], wildlife health or conservation consequences [70], and spillback into susceptible human populations [11].

Our risk assessment is an example of an initial assessment of spillback risk for two pathogens, from which surveillance and management efforts, as well as data deficiencies, can be prioritized. Data on spillback events, by their very nature of being rare and potentially impactful, are the primary limiting factor in the mechanistic understanding of zoonotic pathogen spread, and having an evidence-based sampling guide can be used to guide the sampling needed to reduce the data deficiency gap. With improved data and records of spillback events, we can continue to assess factors that contribute to these events, including transmission pathways, host, and pathogen ecology. For example, are spillback events largely constrained to animals that are considered peridomestic? Or does exposure occur more indirectly with interactions between viral fomites and wildlife? Addressing these questions requires targeted effort and resources including needing to sample high-risk taxa at the appropriate spatial area and the ability to adjust model inputs and evaluate outputs in real-time. Responding to zoonotic pathogens with epidemic potential is challenging; however, this rapid risk assessment model provides a framework from which researchers and managers can efficiently adapt surveillance efforts, mitigation priorities, and management actions.

## Figures and Tables

**Table 1:** To assess the sensitivity of our risk assessment to *how* we quantified our spillback components, we reran our analysis using alternative measurements of the different risk assessment components.

Pathogen	Component	Base metric	Base metric description	Alternative metric	Metric description
SARS-CoV-2	Susceptibility	Majority ranking	Each host susceptibility ranking was assigned based on its majority susceptibility ranking across all reviewed studies	High ranking	Each host susceptibility ranking was based on the highest qualitative susceptibility ranking in the studies we reviewed
				Low ranking	Each host susceptibility ranking was based on the lowest qualitative susceptibility ranking in the studies we reviewed
				Random	Each host susceptibility ranking was assigned randomly
	Exposure	IUCN records	Species distribution was weighed based on estimated range from IUCN	GBIF	Species distribution was weighted based on county-level Global Biodiversity Information Facility (GBIF) records
		WUI-population	Spatial value of intermingling of human population and undeveloped wildland vegetation	Housing	The wildlife urban interface was quantified based on housing instead of the human population
	Introduction	AUC	Introduction pressure quantified as the area under the curve of the temporal trend of infection rate between March 3, 2022 and August 4, 2022	Max	The introduction pressure was quantified using the maximum infection rate between March 3, 2022 and August 4, 2022
				Recent	The introduction pressure was quantified using solely the most recent infection rate data, August 4, 2022

MPOX	Susceptibility	Binomial susceptibility levels	Host susceptibility was assigned based on whether the taxa was known to be susceptible	Three susceptibility levels	Host susceptibility was assigned based on three criteria. High susceptibility was assigned if a taxon was confirmed to be susceptible based on experimental or field-based studies. Medium susceptibility was assigned if a taxon was theoretically susceptible but never confirmed, and low was assigned if taxa were considered not susceptible or not considered at all
				Random	Each host susceptibility ranking was assigned randomly
	Exposure	IUCN records	Species distribution was weighed based on estimated range from IUCN	GBIF	Species distribution was weighted based on county-level Global Biodiversity Information Facility (GBIF) records
		WUI-population	Spatial value of intermingling of human population and undeveloped wildland vegetation	Housing	The wildlife urban interface was quantified based on housing instead of the human population
	Introduction	County population weighed state infection rate	Introduction pressure quantified as the product of the per-person infection level at the state level and the county's total population	State per capita	Introduction pressure was quantified based on state per capita infection level, all counties in a given state have the same values

*Note:* In this table, we define the alternative metrics for each pathogen and spillback process component (susceptibility, exposure, and introduction) combination. Spillback is the spread of infectious diseases from humans into wildlife populations.

Abbreviation: IUCN, International Union for Conservation of Nature database.

## Data Availability

The data generated in this study are available in a U.S. Geological Survey data release [71].

## References

[1] Aguirre A. A., Catherina R., Frye H., Shelley L. (2020). Illicit Wildlife Trade, Wet Markets, and COVID-19: Preventing Future Pandemics. *World Medical & Health Policy*.

[2] Olival K. J., Cryan P. M., Amman B. R. (2020). Possibility for Reverse Zoonotic Transmission of SARS-CoV-2 to Free-Ranging Wildlife: A Case Study of Bats. *PLoS Pathogens*.

[3] Gryseels S., De Bruyn L., Gyselings R., Calvignac-Spencer S., Leendertz F. H., Leirs H. (2021). Risk of Human-to-Wildlife Transmission of SARS-CoV-2. *Mammal Review*.

[4] Montecino-Latorre D., Goldstein T., Gilardi K. (2020). Reproduction of East-African Bats May Guide Risk Mitigation for Coronavirus Spillover. *One Health Outlook*.

[5] Plowright R. K., Reaser J. K., Locke H. (2021). Land Use-Induced Spillover: A Call to Action to Safeguard Environmental, Animal, and Human Health. *The Lancet Planetary Health*.

[6] Graham R. L., Baric R. S. (2010). Recombination, Reservoirs, and the Modular Spike: Mechanisms of Coronavirus Cross-Species Transmission. *Journal of Virology*.

[7] Tao Y., Shi M., Chommanard C. (2017). Surveillance of Bat Coronaviruses in Kenya Identifies Relatives of Human Coronaviruses NL63 and 229E and Their Recombination History. *Journal of Virology*.

[8] Nugent G. (2011). Maintenance, Spillover and Spillback Transmission of Bovine Tuberculosis in Multi-Host Wildlife Complexes: A New Zealand Case Study. *Veterinary Microbiology*.

[9] Rhyan J. C., Spraker T. R. (2010). Emergence of Diseases From Wildlife Reservoirs. *Veterinary Pathology*.

[10] Barron M. C., Nugent G., Cross M. L. (2013). Importance and Mitigation of the Risk of Spillback Transmission of *Mycobacterium bovis* Infection for Eradication of Bovine Tuberculosis From Wildlife in New Zealand. *Epidemiology and Infection*.

[11] Fagre A. C., Cohen L. E., Eskew E. A. (2022). Assessing the Risk of Human-to-Wildlife Pathogen Transmission for Conservation and Public Health. *Ecology Letters*.

[12] Morse S. S., Mazet J. A., Woolhouse M. (2012). Prediction and Prevention of the Next Pandemic Zoonosis. *The Lancet*.

[13] Plowright R. K., Eby P., Hudson P. J. (2015). Ecological Dynamics of Emerging Bat Virus Spillover. *Proceedings of the Royal Society B*.

[14] Plowright R. K., Parrish C. R., McCallum H. (2017). Pathways to Zoonotic Spillover. *Nature Reviews Microbiology*.

[15] Becker D. J., Eby P., Madden W., Peel A. J., Plowright R. K. (2023). Ecological Conditions Predict the Intensity of Hendra Virus Excretion Over Space and Time From Bat Reservoir Hosts. *Ecology Letters*.

[16] Chen S., Bonanno G. A. (2020). Psychological Adjustment During the Global Outbreak of COVID-19: A Resilience Perspective. *Psychological Trauma: Theory, Research, Practice, and Policy*.

[17] Schell D., Wang M., Huynh T. L. D. (2020). This Time Is Indeed Different: A Study on Global Market Reactions to Public Health Crisis. *Journal of Behavioral and Experimental Finance*.

[18] Hartley D. M., Perencevich E. N. (2020). Public Health Interventions for COVID-19: Emerging Evidence and Implications for An Evolving Public Health Crisis. *JAMA*.

[19] Han B. A., Kramer A. M., Drake J. M. (2016). Global Patterns of Zoonotic Disease in Mammals. *Trends in Parasitology*.

[20] Olival K. J., Hosseini P. R., Zambrana-Torrelio C., Ross N., Bogich T. L., Daszak P. (2017). Host and Viral Traits Predict Zoonotic Spillover From Mammals. *Nature*.

[21] Becker D. J., Washburne A. D., Faust C. L. (2019). Dynamic and Integrative Approaches to Understanding Pathogen Spillover. *Philosophical Transactions of the Royal Society B: Biological Sciences*.

[22] Guth S., Hanley K. A., Althouse B. M., Boots M. (2020). Ecological Processes Underlying the Emergence of Novel Enzootic Cycles: Arboviruses in the Neotropics As a Case Study. *PLOS Neglected Tropical Diseases*.

[23] Chandler J. C., Bevins S. N., Ellis J. W. (2021). SARS-CoV-2 Exposure in Wild White-Tailed Deer (*Odocoileus virginianus*). *Proceedings of the National Academy of Sciences*.

[24] Fischhoff I. R., Castellanos A. A., Rodrigues J. P., Varsani A., Han B. A. (2021). Predicting the Zoonotic Capacity of Mammals to Transmit SARS-CoV-2. *Proceedings of the Royal Society B*.

[25] WHO COVID-19: Case Definitions (2022). https://apps.who.int/iris/bitstream/handle/10665/360579/WHO-2019-nCoV-Surveillance-Case-Definition-2022.1-eng.pdf.

[26] Centers for Disease Control and Prevention (2022). COVID Data Tracker. https://covid.cdc.gov/covid-data-tracker.

[27] Ferretti L., Wymant C., Kendall M. (2020). Quantifying SARS-CoV-2 Transmission Suggests Epidemic Control With Digital Contact Tracing. *Science*.

[28] U.S. Department of Agriculture Animal and Plant Health Inspection Service (2020). USDA Statement on the Confirmation of COVID-19 in a Tiger in New York. https://www.aphis.usda.gov/aphis/newsroom/news/sa_by_date/sa-2020/ny-zoo-covid-19.

[29] Larsen H. D., Fonager J., Lomholt F. K. (2021). Preliminary Report of An Outbreak of SARS-CoV-2 in Mink and Mink Farmers Associated With Community Spread, Denmark, June to November 2020. *Eurosurveillance*.

[30] Shriner S. A., Ellis J. W., Root J. J. (2021). SARS-CoV-2 Exposure in Escaped Mink, Utah, USA. *Emerging Infectious Diseases*.

[31] Bae D. Y., Tark D., Moon S. H. (2022). Evidence of Exposure to SARS-CoV-2 in Dogs and Cats From Households and Animal Shelters in Korea. *Animals*.

[32] Di Giulio D. B., Eckburg P. B. (2004). Human Monkeypox: An Emerging Zoonosis. *The Lancet Infectious Diseases*.

[33] Beer E. M., Rao V. B., Holbrook M. R. (2019). A Systematic Review of the Epidemiology of Human Monkeypox Outbreaks and Implications for Outbreak Strategy. *PLoS Neglected Tropical Diseases*.

[34] Kava C. M., Rohraff D. M., Wallace B. (2022). Epidemiologic Features of the Monkeypox Outbreak and the Public Health Response—United States, May 17–October 6 2022. *Morbidity and Mortality Weekly Report*.

[35] Centers for Disease Control and Prevention (2023). MPOX Data Tracker. https://www.cdc.gov/poxvirus/mpox/response/2022/index.html.

[36] Hutson C. L., Lee K. N., Abel J. (2007). Monkeypox Zoonotic Associations: Insights From Laboratory Evaluation of Animals Associated With the Multi-State US Outbreak. *The American Journal of Tropical Medicine and Hygiene*.

[37] Nolen L. D., Osadebe L., Katomba J. (2016). Extended Human-to-Human Transmission During a Monkeypox Outbreak in the Democratic Republic of the Congo. *Emerging Infectious Diseases*.

[38] Parker S., Nuara A., Buller R. M. L., Schultz D. A. (2007). Human Monkeypox: An Emerging Zoonotic Disease. *Future Microbiology*.

[39] Alakunle E. F., Okeke M. I. (2022). Monkeypox Virus: A Neglected Zoonotic Pathogen Spreads Globally. *Nature Reviews Microbiology*.

[40] Dhama K., Khan S., Tiwari R. (2020). Coronavirus Disease 2019-COVID-19. *Clinical Microbiology Reviews*.

[41] MacDiarmid S. C., Pharo H. J. (2003). Risk Analysis: Assessment, Management and Communication. *Revue Scientifique et Technique de l’OIE*.

[42] U.S. Census Bureau (2020). 2020 American Community Survey 3-Year Public Use Microdata Samples.

[43] Goodrich B., Gabry J., Ali I., Brilleman S. (2022). https://mc-stan.org/rstanarm/.

[44] Radeloff V. C., Hammer R. B., Stewart S. I., Fried J. S., Holcomb S. S., McKeefry J. F. (2005). The Wildland-Urban Interface in the United States. *Ecological Applications*.

[45] IUCN (2022). The IUCN Red List of Threatened Species. Version 2022-1. https://www.iucnredlist.org.

[46] Pebesma E. J. (2018). Simple Features for R: Standardized Support for Spatial Vector Data. *The R Journal*.

[47] Medina-Enríquez M. M., Lopez-León S., Carlos-Escalante J. A., Aponte-Torres Z., Cuapio A., Wegman-Ostrosky T. (2020). ACE2: The Molecular Doorway to SARS-CoV-2. *Cell & Bioscience*.

[48] Scialo F., Daniele A., Amato F. (2020). ACE2: The Major Cell Entry Receptor for SARS-CoV-2. *Lung*.

[49] Bernard S., Anderson S. (2006). Qualitative Assessment of Risk for Monkeypox Associated With Domestic Trade in Certain Animal Species, United States. *Emerging Infectious Diseases*.

[50] Reynolds M. G., Doty J. B., McCollum A. M., Olson V. A., Nakazawa Y. (2019). Monkeypox Re-Emergence in Africa: A Call to Expand the Concept and Practice of One Health. *Expert Review of Anti-Infective Therapy*.

[51] Haddad N. (2022). The Presumed Receptivity and Susceptibility to Monkeypox of European Animal Species. *Infectious Diseases Now*.

[52] Carnell R., Carnell M. R. (2016). Package ‘lhs’. https://cran.rproject.org/web/packages/lhs/lhs.pdf.

[53] R Core Team (2022). R: A Language and Environment for Statistical Computing. https://www.R-project.org/.

[54] Bosco-Lauth A. M., Root J. J., Porter S. M. (2021). Peridomestic Mammal Susceptibility to Severe Acute Respiratory Syndrome Coronavirus 2 Infection. *Emerging Infectious Diseases*.

[55] Goldberg A. R., Langwig K. E., Brown K. L. (2024). Widespread Exposure to SARS-CoV-2 in Wildlife Communities. *Nature Communications*.

[56] Brown K., Leggat P. A. (2016). Human Monkeypox: Current State of Knowledge and Implications for the Future. *Tropical Medicine and Infectious Disease*.

[57] Falendysz E. A., Lopera J. G., Rocke T. E., Osorio J. E. (2023). Monkeypox Virus in Animals: Current Knowledge of Viral Transmission and Pathogenesis in Wild Animal Reservoirs and Captive Animal Models. *Viruses*.

[58] Mollentze N., Keen D., Munkhbayar U., Biek R., Streicker D. G. (2022). Variation in the ACE2 Receptor Has Limited Utility for SARS-CoV-2 Host Prediction. *elife*.

[59] Gortázar C., Acevedo P., Ruiz-Fons F., Vicente J. (2006). Disease Risks and Overabundance of Game Species. *European Journal of Wildlife Research*.

[60] Chaves A., Montecino-Latorre D., Alcázar P., Suzán G. (2021). Wildlife Rehabilitation Centers as a Potential Source of Transmission of SARS-CoV-2 Into Native Wildlife of Latin America. *Biotropica*.

[61] Cook J. D., Grant E. H. C., Coleman J. T. H., Sleeman J. M., Runge M. C. (2021). Risks Posed by SARS-CoV-2 to North American Bats During Winter Fieldwork. *Conservation Science and Practice*.

[62] Mueller M. A., Drake D., Allen M. L. (2019). Using Citizen Science to Inform Urban Canid Management. *Landscape and Urban Planning*.

[63] Lopez B., Minor E., Crooks A. (2020). Insights Into Human-Wildlife Interactions in Cities From Bird Sightings Recorded Online. *Landscape and Urban Planning*.

[64] Franklin A. B., Bevins S. N. (2020). Spillover of SARS-CoV-2 Into Novel Wild Hosts in North America: A Conceptual Model for Perpetuation of the Pathogen. *Science of the Total Environment*.

[65] Isaac N. J., Jarzyna M. A., Keil P. (2020). Data Integration for Large-Scale Models of Species Distributions. *Trends in Ecology & Evolution*.

[66] Zipkin E. F., Zylstra E. R., Wright A. D. (2021). Addressing Data Integration Challenges to Link Ecological Processes across Scales. *Frontiers in Ecology and the Environment*.

[67] Kelly D. W., Paterson R. A., Townsend C. R., Poulin R., Tompkins D. M. (2009). Parasite Spillback: A Neglected Concept in Invasion Ecology?. *Ecology*.

[68] Kuchipudi S. V., Surendran-Nair M., Ruden R. M. (2022). Multiple Spillovers From Humans and Onward Transmission of SARS-CoV-2 in White-Tailed Deer. *Proceedings of the National Academy of Sciences*.

[69] Caserta L. C., Martins M., Butt S. L. (2023). White-Tailed Deer (*Odocoileus virginianus*) May Serve as a Wildlife Reservoir for Nearly Extinct SARS-CoV-2 Variants of Concern. *Proceedings of the National Academy of Sciences*.

[70] Leendertz S. A. J., Wich S. A., Ancrenaz M. (2017). Ebola in Great Apes-Current Knowledge, Possibilities for Vaccination, and Implications for Conservation and Human Health. *Mammal Review*.

[71] McDevitt-Galles T., Fry T. L., Richgels K. L. D., Daniel A., Grear D. A. (2025). Data Release for Rapid Risk Assessment Framework to Estimate Potential for Spillback at Human-Wildlife Interfaces: U.S. Geological Survey data release.

